# HPV prevalence in vulvar cancer in Austria

**DOI:** 10.1007/s00508-017-1255-2

**Published:** 2017-09-07

**Authors:** Sophie Pils, Lisa Gensthaler, Laia Alemany, Reinhard Horvat, Silvia de Sanjosé, Elmar A. Joura

**Affiliations:** 1 Department of Obstetrics and Gynecology, Medical University of Vienna, Vienna, Austria; 2 Unit of Infections and Cancer, Cancer Epidemiology Research Program, Catalan Institute of Oncology, IDIBELL, L’Hospitalet de Llobregat, Barcelona, Spain; 3 Department of Pathology, Medical University of Vienna, Vienna, Austria; 4 Department of Gynecology and Gynecologic Oncology, Comprehensive Cancer Center, Medical University of Vienna, Waehringer Guertel 18–20, 1090 Vienna, Austria

**Keywords:** HPV, Vulvar cancer

## Abstract

**Background:**

Even if vulvar cancer is not common, over one hundred women are affected in Austria per year. There is strong evidence that basaloid and warty variants are associated with types of human papillomavirus (HPV).

**Methods:**

The aim of this study is to analyze the types of HPV in vulvar cancer in Austria. This cross-sectional period-prevalence international collaborative study on archival specimens was performed in cooperation with the Institut Catalan di Oncologia in Barcelona, Spain. A total of 177 consecutive samples of Austrian women were analyzed to detect the presence of various HPV types using the SPF10 PCR/DEIA/LiPA25 system. Furthermore, the expression of the tumor suppressor protein p16^INK4a^ was analyzed by immunohistochemistry (CINtec histology kit, ROCHE). A tumor was considered HPV-driven if an overexpression of p16^INK4a^ was detected.

**Results:**

In all, 41 cases of vulvar cancer tested positive for HPV DNA (23%) and 32 (18%) were p16 positive. Patients with warty and basaloid squamous cell cancer were significantly younger than those with keratinizing squamous cell cancer (63.3 years vs. 71.0 years, *p* = 0.021). In addition, 77.4% of all cases suffering from warty or basaloid squamous cell cancer tested positive for HPV, compared to 9.5% of the keratinizing squamous cell cancer cases (*p* < 0.001). The most commonly detected HPV strain was type 16, followed by 31 and 33.

**Conclusion:**

Infection with HPV type 16 appears to be strongly correlated to the development of warty or basaloid squamous cell cancer. Vaccination against HPV can be expected to prevent this type of vulvar cancer.

## Introduction

In Europe, the incidence of vulvar cancer has ranged between 2.9 and 4.1 cases per 100,000 women over the last few decades. Due to the fact that this disease mostly affects women above the age of 60, this rate is age-adjusted. In Austria, the number of newly diagnosed cases per year has been between 114 and 163 in the period from 1990 to 2010. Vulvar cancer is thus a rare disease [1].

Over the past few decades, the incidence rates of vulvar intraepithelial neoplasia (VIN) and invasive vulvar cancer (IVC) have both been reported to have increased in Austria, in other European countries and worldwide, particularly among younger women [2–6]. Squamous cell carcinoma accounts for more than 90% of the malignant tumors of the vulva. Basaloid and warty variants associated with regular type VIN are more common in younger women and are associated with human papillomavirus (HPV). In contrast, keratinizing variants arising from chronic vulvar dermatosis, such as lichen sclerosus, and associated with differentiated VIN but not with HPV occur in older women [7].

We present the Austrian data of an international collaborative study to evaluate the HPV contribution and genotype distribution in vulvar lesions from pathological archives in 39 countries from five continents [8]. The primary objective was to describe the HPV DNA prevalence and type distribution in HPV-related vulvar cancer cases. The secondary objective was to describe the HPV prevalence and HPV type distribution in vulvar cancers by main histological groups, age at diagnosis and year of diagnosis and the correlation between HPV testing and p16^INK4a^ in cancers of the vulva.

## Materials and methods

The cross-sectional period-prevalence study on archival specimens included a common standard protocol and a sensitive assay was used for HPV-DNA detection (SPF10/DEIA/LiPA25 system). IVC cases were further tested for the cyclin-dependent kinase-4 inhibitor (p16^INK4A^) that is reported to be overexpressed in at least 90% of VIN- and HPV-related IVC cases [9–12].

A total of 204 consecutive samples was sent to the study center at the Institut Catalan di Oncologia (ICO) at Barcelona, Spain. Of those, 16 were submitted as control specimens according to the protocol. The remaining 188 cases of invasive vulvar cancers were conserved in paraffin blocks at the Medical University of Vienna between 1994 and 2010. The tissue material was re-embedded at the ICO if the paraffin block did not satisfy the requirements of the study. Microtome sectioning of the specimens under noncontamination conditions and sandwich technique were carried out to confirm an optimal number of sections to be used for DNA extraction, HPV-DNA detection, and HPV genotyping. First and last sections were hematoxylin–eosin stained for pathological review and the in-between sections were used for the HPV-DNA detection. All cases were reviewed by a trained pathologist at the ICO to assess the diagnosis and quality of the specimen before HPV testing. A case was considered suitable for HPV-DNA testing when tumoral cells were found in the two hematoxylin–eosin slides. Cases difficult to classify, cases with a dissenting diagnosis compared to the original one and all the rare histological types were further reviewed by two senior expert pathologists at the ICO. DNA was extracted under noncontamination protocols and aliquoted. HPV testing was performed on each specimen using the SPF-10 broad spectrum primers PCR followed by DNA enzyme immunoassay (DEIA). HPV-DNA-positive samples were subsequently analyzed by SPF10 PCR/DEIA/LIPA25 (RHA Kit HPV SPF10-LiPA25, version 1 by Labo Bio-medical Products, Rijswijk, The Netherlands), a reverse hybridization technique that detects 25 high-risk (HR) and low-risk (LR) HPV types (6, 11, 16, 18, 31, 33, 34, 35, 39, 40, 42, 43, 44, 45, 51, 52, 53, 54, 56, 58, 59, 66, 68, 70, 74). p16^INK4A^ was performed following manufacturer’s instructions (CINtec® PLUS Cytology Kit by Roche) in the invasive vulvar cancer cases. A case was considered to be positive if more than 25% of invasive cancer cells showed a diffuse overexpression. External audit from the reference laboratory and from the Steering Committee of the Study was done periodically. A committee of pathologists monitored the study periodically. The protocol was approved by local and the ICO ethics committees [8].

## Results

Of the 188 analyzed cases, 7 were excluded according to protocol and 4 cases were not verified as invasive disease. For this analysis, 177 cases of IVC remained. These can be divided into 31 cases of warty or basaloid squamous cell cancer (SCC) and 126 cases of keratinizing SCC, as well as 18 mixed and 2 other cases (one adenocarcinoma not otherwise specified and one basocellular carcinoma) (Table 1).

**Table 1:** Invasive vulvar cancer cases from Austria, stratified by histological type

Characteristic	Invasive vulvar cancer cases		Histological groups							
			SCC Warty/Basaloid		SCC Keratinizing		SCC Mixed		Other^a^	
**Socio-demographic variables**										
*Age*										
Mean (st.dev.)	69.8	(13.9)	63.3	(17.1)	71.0	(13.0)	73.8	(10.5)	63.5	(23.3)
Range (min–max)	66	(31–97)	66	(31–97)	58	(34–92)	45	(48–93)	33	(47–80)
*ANOVA test P‑value*	–		***0.0208***							
**HPV detection variables**	*N*	%	*N*	%	*N*	%	*N*	%	*N*	%
*HPV DNA detection*										
Negative	136	77	7	23	114	90	13	72	2	100
Positive	41	23	24	77	12	10	5	28	–	–
*Fisher’s exact test P‑value*	–		***<0.001***							
*p16* ^*INK4a*^ * expression* ^*b*^										
Negative	138	78	11	35	114	90	12	67	1	50
Positive	38	22	20	65	11	9	6	33	1	50
*Fisher’s exact test P‑value*	–		***<0.001***							
**TOTAL**	**177**	**100**	**31**	**18**	**126**	**71**	**18**	**10**	**2**	**1**

*SCC* squamous cell carcinoma, *N* number of invasive vulvar cancer cases, *%* column percent, *HPV* human papilloma virus

^a^1 adenocarcinoma not otherwise specified and 1 basocellular carcinoma

^b^1 p16INK4a not performed due to unavailable material

The average age of all examined patients was 69.8. However, patients with warty and basaloid SCC were significantly younger than those with keratinizing SCC (63.3 years vs. 71.0 years, *p* = 0.021) (Table 1). Among the examined cases, 41 were HPV positive (23%). Overall, IVC cases that were HPV positive were significantly younger (63.9 years vs. 71.6 years, *p* = 0.002) (Table 2).

**Table 2:** Age of invasive vulvar cancer cases according to histological type and HPV positivity

	Invasive vulvar cancer cases		Histological groups							
			SCC Warty/Basaloid		SCC Keratinizing		SCC Mixed		Other^a^	
HPV	Neg	Pos	Neg	Pos	Neg	Pos	Neg	Pos	Neg	Pos
*Age*										
Mean	71.6	63.9	65.7	62.5	71.9	62.8	73.9	73.4	63.5	–
Standard deviation	12.4	17.8	16.6	17.5	12.0	19.1	7.9	16.9	23.3	–
*ANOVA test P‑value*	*0.002*		***0.673***		***0.020***		***0.928***		*–*	

*SCC* squamous cell carcinoma, *Neg* negative, *Pos* positive

^a^1 adenocarcinoma not otherwise specified and 1 basocellular carcinoma

Overall, 77% of all cases suffering from warty or basaloid SCC were HPV positive, compared to 10% of the keratinizing SCC cases (*p* < 0.001). In addition, 38 cases of the 41 HPV-positive cases exhibited a single infection. The most common type was HPV 16 (31/38), followed by HPV 31, 33, and 44 (2/38 each). This distribution can also be observed when focusing only on cases of warty or basaloid SCC. Cases of keratinizing SCC could be linked to HPV 16 (8/10) and HPV 33 and 74 (1/10 each) (Table 3). Overall, 32 (18%) cases were HPV DNA and p16^INK4a^ positive (Table 4). Six (4%) of the 136 HPV-negative cases expressed p16^INK4a^. Among HPV-positive cases, it seems that keratinizing tumors are less p16^INK4a^ positive than warty or basaloid ones. Finally, 88% of the p16^INK4a^-positive cases exhibited HPV 16 (Table 4).

**Table 3:** HPV type distribution of invasive vulvar cancer cases from Austria, stratified by histological groups

HPV type	Invasive vulvar cancer cases HPV/DNA positive		Histological groups					
			SCC Warty/Basaloid		SCC Keratinizing		SCC Mixed	
	*N*	%	*N*	%	*N*	%	*N*	%
*Single HPV infections*	38	93	23	96	10	83	5	100
HPV16	31	76	18	75	8	67	5	100
HPV31	2	5	2	8	–	–	–	–
HPV33	2	5	1	4	1	8	–	–
HPV44	2	4	2	8	–	–	–	–
HPV74	1	2	–	–	1	–	–	–
*Multiple HPV infections*	2	5	1	4	1	8	–	–
HPV16&HPV33	1	2	1	4	–	–	–	–
HPV42&HPV70	1	2	–	–	1	8	–	–
*HPVX*	1	2	–	–	1	8	–	–
*TOTAL*	*41*	*100*	*24*	*100*	*12*	*100*	*5*	*100*

*HPV* human papillomavirus, *N* number of invasive vulvar cancer cases, *%* column percent, *HPVX* DEIA-positive but LiPA25 negative, *SCC* squamous cell carcinoma

**Table 4:** HPV type distribution of invasive vulvar cancer cases from Austria among p16^INK4a^ positive, stratified by histological groups

HPV type	Invasive vulvar cancer cases p16^INK4a^ positive^a^		Histological groups					
			SCC Warty/Basaloid		SCC Keratinizing		SCC Mixed	
	*N*	%	*N*	%	*N*	%	*N*	%
*Single HPV infections*	30	94	18	95	7	88	5	100
HPV16	28	88	17	90	6	75	5	100
HPV31	1	3	1	5	–	–	–	–
HPV33	1	3	–	–	1	12	–	–
*Multiple HPV infections*	2	6	1	5	1	12	–	–
HPV16&HPV33	1	3	1	5	–	–	–	–
HPV42&HPV70	1	3	–	–	1	12	–	–
*TOTAL*	*32*	*100*	*19*	*100*	*8*	*100*	*5*	*100*

*HPV* human papillomavirus, *N* number of cases, *%* column percent, *SCC* squamous cell carcinoma

^a^7 invasive vulvar cancer cases were p16NK4a positive but HPV DNA negative

## Discussion

This analysis confirms the predominant contribution of HPV 16 to the etiology of HPV-positive vulvar cancer and suggests that other HPV types, such as HPV 33 and HPV 31, which are common in cervical cancer and precancer, also play a role in vulvar carcinogenesis although to a much lesser extent. In contrast to the global results, there was no case related to HPV 18 or 45 observed in the Austrian series. A major contribution of this study is that p16^INK4a^ positivity was included in the criteria to consider a tumor to be HPV driven. p16^INK4a^ is reported to be overexpressed in at least 90% of VIN- and HPV-related IVC cases [9–12].

HPV-DNA detected without over-expression of p16^INK4A^ could be a reflection of a transient infection with no role in carcinogenesis. The details of the methods have been published previously [8].

The presented data show an important inverse association of age with HPV prevalence in vulvar cancer tissue. HPV-positive cases with keratinizing vulvar carcinomas were significantly younger, whereas no significant age difference was observed in the other histological subtypes.

In all, 23% of our cases were HPV positive, 18% due to p16^INK4a^ positivity excluding a possible transient infection. This is in accordance with the report of vulvar intraepithelial and invasive neoplasms in Austria [2]. In the global study analyzing 587 cases of VIN and 1709 IVC, HPV-DNA was detected in 86.7 and 28.6% of the cases, respectively. Among IVC cases, 25.1% were both HPV-DNA and p16^INK4A^ positive [8]. Furthermore, we could demonstrate in a previous publication that vagina and cervix are also affected by HPV-related precancer and neoplasms. In our published data, half of the invasive vaginal cancers were reported to be HPV positive with a trend to better survival in the HPV-positive cohort [13]. In a recent global analysis of 408 invasive vaginal cancers, 64% were both HPV and p16^INK4a^ positive. Vaginal neoplasms are also dominated by HPV 16 [14]. We also could demonstrate that HPV 16, 33, and 31 are the most common HPV types in precancerous lesions of the cervix [15, 16].

To conclude, in Austria HPV contributed to approximately a quarter of invasive vulvar cancers. HPV 16 was present in about three-quarters of all HPV positive cases. HPV vaccines may reduce a quarter of IVC based on the reported efficacy of the trial [17, 18]. The nonavalent HPV vaccine is expected to eradicate 97% of the HPV-related IVC [19]. The vaccine is safe and cost effective [20, 21]. Knowledge of noncervical disease is still poor in most patients [22]. In contrast to cervical cancer, no screening programs for vulvar cancers are in place; however, detection of HPV 16 at the cervix may identify women at risk [23].
